# Effects of coenzyme Q10 supplementation on oxidative stress biomarkers following reperfusion in STEMI patients undergoing primary percutaneous coronary intervention

**DOI:** 10.34172/jcvtr.2023.31817

**Published:** 2023-12-30

**Authors:** Amirhossein Yazdi, Kimia Shirmohammadi, Erfan Parvaneh, Taher Entezari-Maleki, Seyed Kianoosh Hosseini, Akram Ranjbar, Maryam Mehrpooya

**Affiliations:** ^1^Department of Cardiology, School of Medicine, Clinical Research Development Unit of Farshchian Hospital, Hamadan University of Medical Sciences, Hamadan, Iran; ^2^Department of Clinical Pharmacy, School of Pharmacy, Medicinal Plants and Natural Products Research Center, Hamadan University of Medical Sciences, Hamadan, Iran; ^3^Department of Clinical Pharmacy, Faculty of Pharmacy, Tabriz University of Medical Sciences, Tabriz, Iran; ^4^Department of Pharmacology Toxicology, School of Pharmacy, Hamadan University of Medical Sciences, Hamadan, Iran

**Keywords:** Acute myocardial infarction, Primary angioplasty, Coenzyme Q10, Reperfusion injury, Oxidative stress, Antioxidants

## Abstract

**Introduction:**

It is well-established that oxidative stress is deeply involved in myocardial ischemia-reperfusion injury. Considering the potent antioxidant properties of coenzyme Q10 (CoQ10), we aimed to assess whether CoQ10 supplementation could exert beneficial effects on plasma levels of oxidative stress biomarkers in patients with ST-elevation myocardial infarction (STEMI) undergoing primary percutaneous coronary intervention (PPIC).

**Methods:**

Seventy patients with the first attack of STEMI, eligible for PPCI were randomly assigned to receive either standard treatments plus CoQ10 (400 mg before PPCI and 200 mg twice daily for three days after PPCI) or standard treatments plus placebo. Plasma levels of oxidative stress biomarkers, including superoxide dismutase (SOD), catalase (CAT), glutathione peroxidase (GPx), total antioxidant capacity (TAC), and malondialdehyde (MDA) were measured at 6, 24, and 72 hours after completion of PPCI.

**Results:**

The changes in plasma levels of the studied biomarkers at 6 and 24 hours after PPCI were similar in the both groups (*P* values>0.05). This is while at 72 hours, the CoQ10- treated group exhibited significantly higher plasma levels of SOD (*P* value<0.001), CAT (*P* value=0.001), and TAC (*P* value<0.001), along with a lower plasma level of MDA (*P* value=0.002) compared to the placebo-treated group. The plasma activity of GPX showed no significant difference between the groups at all the study time points (*P* values>0.05).

**Conclusion:**

This study showed that CoQ10 has the potential to modulate the balance between antioxidant and oxidant biomarkers after reperfusion therapy. Our results suggest that CoQ10, through its antioxidant capacity, may help reduce the reperfusion injury in ischemic myocardium.

## Introduction

 Despite a progressive and gradual decrease in its incidence, acute myocardial infarction (AMI) remains a major cause of short- and long-term mortality worldwide.^1^ Nowadays, timely and successful reperfusion and restoration of blood flow to the ischemic myocardium, achieved through either thrombolytic therapy or primary percutaneous coronary intervention (PPCI), stands as the most effective and well-established therapeutic approach for salvaging ischemic myocardium from impending infarction.^2^ However, despite the indisputable benefits of myocardial reperfusion therapy, it can itself induce additional myocardial injury, known as myocardial ischemia-reperfusion injury, which can paradoxically reduce the beneficial effects of myocardial reperfusion therapy.^3^ At present, the treatment of reperfusion injury following ischemia is primarily supportive, and there is no specific therapeutic approach for preventing ischemia-reperfusion injury in reperfused AMI patients.^4^

 Although the precise molecular mechanism of ischemia-reperfusion injury has not been completely understood, it appears to be multifactorial, and multiple pathological processes, including mitochondrial damage, oxidative stress injury, inflammation, calcium overload, and iron mobilization are involved in its pathogenesis.^5^ The restoration of blood flow and re-oxygenation after a period of ischemia trigger a burst of reactive oxygen species (ROS) generation, disrupting the balance between oxidants and antioxidants in cardiomyocyte and leading to myocardial damage.^6^ Furthermore, cellular oxidative damage and excessive ROS production trigger and activate excessive inflammatory responses, exacerbating cardiomyocyte damage.^7^ This is supported by evidence from both experimental and clinical studies, demonstrating a significant increase in oxidative stress status and a significant decrease in enzymatic and non-enzymatic components of antioxidant defense systems in both plasma and heart tissue following myocardial reperfusion.^8,9^ As such, it is suggested that therapeutic interventions capable of inhibiting ROS production, accelerating ROS consumption, or enhancing the activity of endogenous antioxidant systems have the potential to reduce oxidative stress-induced injury during ischemia and reperfusion. Encouraging results have been achieved, especially in experimental studies.^10,11^

 Coenzyme Q10 (CoQ10) is a lipid-soluble, vitamin-like, ubiquitous compound, acting as an electron transporter for the production of adenosine triphosphate (ATP).^12^ Beyond its primary role in cellular energy supply, CQ10, as a mitochondria-targeted antioxidant, has important roles in protecting cells against oxidative damages.^13,14^ Besides that, CoQ10 also exhibits anti-inflammatory effects,^15^ regulates intracellular calcium hemostasis,^16^ reduces apoptotic cell death,^17^ and improves endothelial function.^18^ High amount of CoQ10 is found in organs with high-energy demands or metabolic activity, such as heart, kidney, liver, and muscle.^19^ Conversely, diminished CoQ10 levels have been reported during myocardial ischemia and reperfusion, correlating with worse clinical outcomes post-myocardial infarction.^20,21^ Numerous high-quality animal studies have consistently shown that treatment with CoQ10 treatment can significantly prevent or attenuate myocardial damage associated with ischemia-reperfusion injury, ultimately leading to improved cardiac function.^22-24^ Based on these promising experimental findings, the potential cardioprotective effects of CoQ10 against myocardial ischemia-reperfusion injury have been explored in some clinical research.^25-27^ For example,, a study conducted on patients undergoing coronary artery bypass graft (CABG) surgery revealed that pretreatment with intravenous CoQ10 effectively prevents left ventricular depression during early reperfusion and minimizes myocardial cellular damage.^25^ Another study found that early administration of CoQ10 to AMI patients with reduced left ventricular ejection fraction is associated with a reduction of left ventricular remodeling following AMI.^26^ It is thought that CoQ10 exerts its cardioprotective effects against ischemia-reperfusion injury through various mechanisms, including ameliorating proinflammatory and oxidative stress responses, improving endothelial function, restoring intracellular calcium hemostasis, and decreasing cellular apoptosis.^28^ Although CoQ10’s impact is multifaceted, its predominant cardioprotective effect is attributed to its robust antioxidant properties.^28^ In the context of coronary artery diseases, a recent systematic review and meta-analysis of randomized controlled trials demonstrated that CoQ10 supplementation improves antioxidant defense systems and ameliorates oxidative stress in patients suffering from coronary artery diseases.^29^ However, to date, there has been no study specifically evaluating the effects of CoQ10 supplementation in patients with ST-segment elevation myocardial infarction (STEMI) undergoing PPCI. Hence, we designed and conducted a study to assess the potential benefits of CoQ10 supplementation in STEMI patients undergoing PPCI. In our previous publication, we reported a portion of findings related to the impact of CoQ10 on myocardial perfusion and myocardial ischemia-reperfusion injury.^30^ In the present study, our specific focus was on the impact of CoQ10 supplementation on plasma levels of oxidative stress after reperfusion therapy.

## Materials and Methods

###  Study design

 This study was a part of a larger double-blind, randomized, parallel-group, placebo-controlled study conducted between November 2021 and August 2022 in a cardiology referral hospital located in West of Iran. The study was conducted in accordance with the principles of the 2013 Declaration of Helsinki and was approved by the Research Ethics Committee of the Hamadan University of Medical Sciences (ethical license: IR.UMSHA. REC.1400.623). The trial was registered in the Iranian Registry of Clinical Trials (www.irct.ir/trial/60026) under trial Number: IRCT20120215009014N407. Participants were informed about the purpose of the study, and written informed consent was obtained in the emergency room before enrolment. Detailed methodological aspects of the study have been previously described in our earlier publication. ^30^

###  Patient enrollment

 Adults patients aged between 18 and 80 years, experiencing their first STEMI, arriving at the hospital within 12 hours of STEMI onset, and scheduled for PPCI were screened for participation in the trial. The diagnosis of STEMI was based on a history of typical chest pain for > 30 min but < 12 h in association with ST-segment elevation ≥ 1 mm in at least two extremities or ≥ 2 mm in at least two precordial electrocardiographic leads or new onset of left bundle branch block.^31^ Patients with any of the following characteristics were excluded: receiving fibrinolytic therapy, experiencing cardiac arrest or arrhythmia requiring cardiopulmonary resuscitation, presence of cardiogenic shock, history of previous myocardial infarction, history of previous percutaneous coronary intervention or coronary artery bypass grafting (CABG), pre-existing renal impairment (defined as estimated glomerular filtration rate [eGFR] ⩽60 mL/min/l.73m2), acute or chronic hepatic dysfunction (LFT’s > 3 × upper limit), acute or chronic inflammatory or infective diseases, history of malignancy, existence of a life-threatening disease with a life expectancy of < 6 months, use any antioxidant and anti-inflammatory supplements other than study medications, use of CoQ10 supplements during one month before the procedure, history of hypersensitivity to contrast media, known CoQ10 allergy, pregnancy or lactation, incapacity or inability to provide informed consent.

 Baseline characteristics, including demographic and clinical characteristics, cardiovascular risk factors, and medical history, as well as initial vital signs, ECG and laboratory data at admission were gathered from medical records and patient interviews. Information on medication use before and after PPCI, time variables including pain-to-balloon time and door-to-balloon time, and angiographic and procedural characteristics were also recorded for each patient.

###  Intervention

 Seventy patients were randomly assigned to the CoQ10 or the placebo group. The oral CoQ10 capsules utilized in this study were manufactured by Bonyan Salamat Kasra Pharmaceutical Company, Tehran, Iran. Identical placebo capsules, containing starch as the inert substance, were prepared by the Department of Pharmaceutics at the School of Pharmacy, Hamadan University Medical Sciences. In the intervention group, patients received oral CoQ10 capsules at a loading dose of 400 mg immediately before the procedure, followed by 200 mg twice daily with meals for three consecutive days after the procedure. Patients in the control group received placebo capsules following the same schedule as the CoQ10 group. The selected dose was based on previous research indicating that an average of 200 mg of oral CoQ10 twice daily, taken with a meal, is necessary to achieve a therapeutic blood level of CoQ10 ( > 2.5 mcg/mL).^32,33^

 Coronary angiography and the stent implantation were performed through a radial or femoral approach, following standard clinical practice.^2^ The decision to use glycoprotein IIb/IIIa inhibitors and thrombus aspiration during the procedure was left to the discretion of the interventionist, based on clinical and angiographic characteristics. All patients received a loading dose of aspirin (325 mg) and ticagrelor (180 mg) or clopidogrel (600 or 300 mg if already received clopidogrel) orally before the procedure. Intravenous heparin (70 IU/kg body mass) was administered to anticoagulate all patients during the procedure, with additional doses guided by the activated clotting time. The standard treatment after PPCI, to prevent subsequent episodes of AMI and adverse left ventricular remodeling, including aspirin, clopidogrel or ticagrelor, angiotensin-converting enzyme inhibitors (ACEIs), or angiotensin receptor blockers (ARBs), beta-blockers, and statins were prescribed to all patients unless contraindicated.

###  Trial registration

 The trial was registered at the Iranian Registry of Clinical Trials (www.irct.ir/trial/60026); identifier code: IRCT20120215009014N407; registration date: 2021-11-18.

###  Measurements of serum biochemical markers

 To evaluate changes in plasma levels of oxidative stress biomarkers following reperfusion therapy, approximately 5 mL venous blood samples were collected from each patient at four time points: before the procedure and at 6, 24, and 72 hours after the procedure. After centrifugation at 3000 rpm for 10 minutes, separated serum samples were stored at −70 °C until analyses. All biomarkers were analyzed in duplicate, with the variations of repeated determinations being within 10% of the same sample. The response of the endogenous enzymatic antioxidant defense system was assessed by measuring the plasma activity of superoxide dismutase (SOD), catalase (CAT), and glutathione peroxidase (GPx). The plasma SOD activity was quantified spectrophotometrically using the modified nitrite method, as previously described by Oyanagui.^34^ SOD was expressed as U/mL. The activity of CAT in plasma was determined by measuring the decrease in hydrogen peroxide concentration at 230 nm, following the Beutler’s method.^35^ CAT activity was expressed as U/mL. The plasma GPX activity was measured spectrophotometrically using the method described by Flohe and Gunzler.^36^ This method is based on the oxidation of Glutathione (GSH) by GPx in the presence of hydrogen peroxide, resulting in the production of glutathione disulfide (GSSG), and the activity was expressed as mU/mL The plasma level of Total Antioxidant Capacity (TAC) was used as a marker of non-enzymatic antioxidant cellular defense systems. The plasma TAC concentration was determined using Ferric Reducing Antioxidant Power technique (FRAP), in which a colorless ferric tripyridyltriazine complex is reduced to a blue ferrous complex by the antioxidants in the serum.^37^ The plasma malondialdehyde (MDA) level, indicating oxidative stress status, was determined spectrophotometrically using the thiobarbituric acid reactive substances (TBARs) method, as described by Botsoglou et al.^38^

###  Study endpoints

 As the primary endpoint, we compared changes in plasma levels of SOD, GPX, CAT, TAC, and MDA at 6, 24, and 72 hours post-procedure between the study groups. Secondary endpoints were the occurrence of short-term major adverse cardiovascular events (MACE), including all-cause mortality, cardiovascular mortality, nonfatal myocardial infarction, revascularization of the culprit artery, hospitalization due to heart failure (HF), and occurrence of cerebrovascular events during the 28-day follow-up. The occurrence of patients’ MACE was followed through a combination of phone calls and scheduled face-to-face follow-up visits.

###  Sample Size Calculation

 Given that there was no prior study investigating the effect of CoQ10 supplementation on oxidative stress biomarkers during myocardial reperfusion in patients with STEMI, for estimating the sample size, a pilot study was conducted. In this pilot study, 4 patients were studied in each group. According to the results of the pilot study, the mean (SD) plasma level of MDA was 2.15 (0.422) in the intervention group and 2.43 (0.32) in the control groups after 72 hours of PPCI. Based on these results, we arrived at a sample size of 35 for each group and a total sample size of 70, with a power of 80%, a two-sided α of 0.05, and an estimated dropout rate of 10%.

###  Randomization and blinding 

 Stratified patients were randomly assigned into the CoQ10 and placebo groups using the block randomization method. Placebo capsules were similar to CoQ10 capsules in size, color, shape, and packaging. Both the investigators and patients were blinded to the assigned treatment allocation until the statistical analyses were completed.

###  Statistical analysis

 Data analysis was performed by the SPSS software (Version 20.0; IBM Corp., Armonk, N.Y., USA). All analyses followed the intention-to-treat principle, with the missing values replaced by baseline values of the outcome variables. Continuous variables were expressed as the mean ± standard deviation, and categorical variables were presented as frequency (percentage). Student’s t test was also used for the comparison of quantitative data between the study groups. Categorical variables were compared using chi-square statistics, or Fischer’s exact test, as appropriate. Serial levels of biomarkers were compared within and between groups using repeated-measures analysis of variance (ANOVA). Differences between groups were considered significant at *P* values ≤ 0.05.

## Results

###  Demographics and baseline characteristics

**Figure 1:**
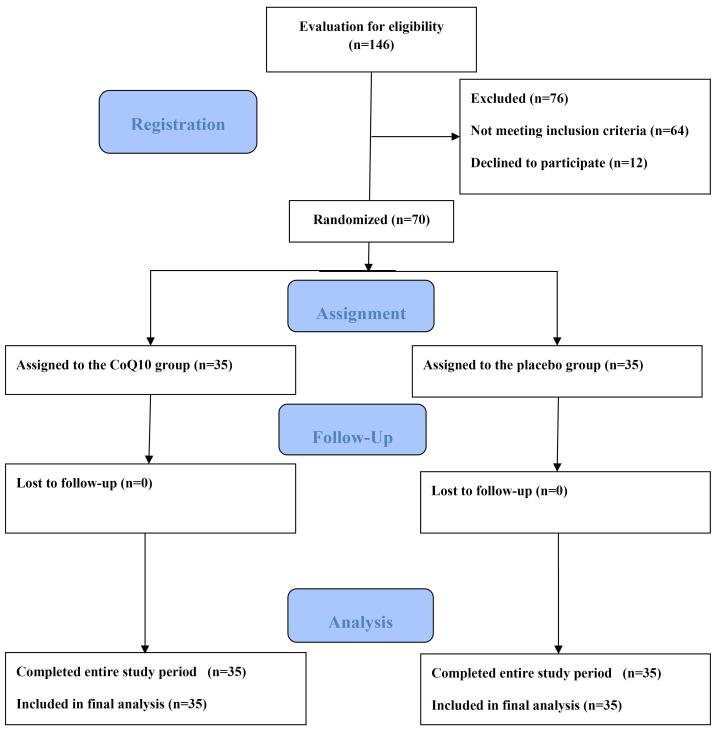
Flow chart showing the inclusion of patients in the study

 The trial’s flowchart is depicted in Figure 1. Between November 2021 and August 2022, a total of 146 consecutive patients diagnosed with their first ST-elevation myocardial infarction (STEMI) and eligible for PPCI underwent screening for potential participation in the trial. Out of these,76 patients were excluded: 64 due to not meeting eligibility criteria and 12 who declined to participate. Ultimately, 70 patients, with 35 assigned to the CoQ10 group and 35 to the placebo group, were included in the trial. All participants successfully completed a 3-day treatment protocol and were included in the final analysis. The depiction of participant progression through different stages of the research process can be also found in Figure 1 of our previous publication.^30^

Table 1 summarized the key demographic data and clinical features of the study groups. The two groups exhibited comparable baseline characteristics. The mean age of the precipitants was 63.25 ± 7.94 years, with an age range of 45 to 75 years. Of the total, 50 patients (71.4%) were male, and 20 patients (28.6%) were female. The medical history and risk factors for coronary artery disease were comparable between the study groups. No significant differences were noted between the groups in terms of medication use both before and after admission. Additionally, there was no difference between the groups in terms of mean pain-to-balloon time, mean door-to-balloon time, and the distribution of the culprit artery. A majority of the procedures in both groups were conducted via radial access, and drug-eluting stents were implanted in all study patients. The frequency of patients receiving Glycoprotein IIb/IIIa inhibitors or thrombus aspiration was also similar between the groups. Detailed demographic data and clinical features of the study groups are presented in Table 1 of our previous publication.^30^

**Table 1:** Comparison of demographic and baseline clinical information of the study groups

**Parameters**	**Control Group (n=35) **	**CoQ10 Group (n=35) **	***P*** ** value**
Gender, male/female, n(%)	24/11(68.6/31.4)	26/9 (74.3/25.7)	0.792^†^
Body mass index, (kg/m^2^)	24.58 ± 2.50	24.75 ± 3.31	0.814^*^
Age, (years)	64.65 ± 7.45	61.85 ± 8.27	0.142^*^
**Cardiovascular risk factors, **n (%)			
Family history of coronary artery disease	11 (31.40)	8 (22.90)	0.592^†^
Smoking	16 (45.70)	11 (31.40)	0.326^†^
Dyslipidemia	20 (57.10)	21(60.0)	1.000^†^
Diabetes mellitus	9 (25.70)	8 (22.90)	1.000^†^
Hypertension	18 (51.40)	20 (57.10)	0.811^†^
Door-to-balloon time, (min)	42.57 ± 5.98	42.71 ± 6.34	0.923^*^
Pain-to-balloon time, (min)	245.85 ± 34.54	242.28 ± 35.83	0.673^*^
**Culprit lesion, n (%)**			0.900^†^
Left main	0 (0.0)	1 (2.90)	
Left anterior descending	16 (45.70)	16 (45.70)	
Left circumflex	5 (14.30)	6 (17.10)	
Right coronary artery	14 (40.0)	12 (34.30)	
SBP, (mmHg)	126.11 ± 10.57	123.54 ± 7.26	0.240^*^
DBP, (mmHg)	72.40 ± 5.04	73.22 ± 6.06	0.536^*^
Left ventricular ejection fraction,%	50.42 ± 7.22	51.85 ± 8.18	0.442^*^
Heart rate, Mean ± SD	77.87 ± 11.35	79.34 ± 12.47	0.611^*^
**Baseline biochemical assessments**			
TC, (mmol/L)	5.72 ± 1.25	5.65 ± 0.99	0.814^*^
TG, (mmol/L)	1.84 ± 0.42	1.88 ± 0.60	0.795^*^
LDL-C, (mmol/L)	3.53 ± 0.44	3.31 ± 0.66	0.748^*^
HDL-C, (mmol/L)	0.98 ± 0.14	0.99 ± 0.17	0.774^*^
FBS, (mg/dL**)**	6.75 ± 1.15	6.61 ± 1.07	0.580^*^
Serum creatinine, (mg/dL)	0.99 ± 0.09	1.05 ± 0.23	0.174^*^
CK-MB, (ng/mL)	52.42 ± 7.47	47.74 ± 6.85	0.080^*^
Troponin I, (ng/mL)	5.31 ± 4.59	6.61 ± 7.68	0.392^*^
**Pharmacological treatment before admission**, n(%)			
Angiotensin-converting-enzyme inhibitors	13 (37.10)	11 (31.40)	0.802^†^
Angiotensin-receptor blockers	5 (14.30)	6 (17.10)	1.000^†^
Diuretics	10 (28.60)	9 (25.70)	1.000^†^
Anti-platelet agents	15 (42.90)	12 (34.30)	0.624^†^
Calcium-channel blockers	8 (22.90)	7 (20.00)	1.000^†^
Statins	13 (37.10)	9 (25.70)	0.440^†^
Beta blockers treatment	12 (34.30)	10 (28.60)	0.797^†^
Oral anti-diabetic agents	2 (5.70)	4 (11.40)	0.673^†^
Insulin	5 (14.30)	3 (8.60)	0.710^†^
Combination of Insulin and oral anti-diabetic agents	2 (5.70)	1(2.90)	1.000^†^
**Pharmacological treatment during hospitalization**, n(%)			
Aspirin	35 (100)	35 (100)	1.000^†^
Ticagrelor	22 (62.90)	24 (68.60)	0.802^†^
Clopidogrel	13 (37.10)	11 (31.40)	0.802^†^
Angiotensin-converting-enzyme inhibitors	28 (80.0)	26 (74.30)	0.777^†^
Angiotensin-receptor blockers	7 (20. 0)	9 (25.70)	0.777^†^
Beta-blockers	31 (88.60)	30 (85.70)	1.000^†^
Calcium-channel blockers	6 (17.10)	4 (11.40)	0.734^†^
Statins	35 (100)	35 (100)	1.000^†^
Nitrates	7 (20.0)	6 (17.10)	1.000^†^
Diuretics	4 (11.4)	7 (20.0)	0.513^†^
Spironolactone/eplerenone	7 (20.0)	10 (28.60)	0.578^†^

Continuous data were presented as means ± SD; TC: Total cholesterol; TG: Triglyceride; LDL-C: low density lipoprotein-cholesterol; HDL-C: high density lipoprotein-cholesterol; CoQ10 = Coenzyme Q10; SBP: systolic blood pressure; DBP: systolic blood pressure; CK-MB: creatine kinease myocardial band isoenzyme. Data were analyzed using the student t-test (^*^) or chi square (^†^) test. *P* < 0.05 statistically significant.

###  Comparison of oxidative stress biomarkers between the two groups

**Figure 2:**
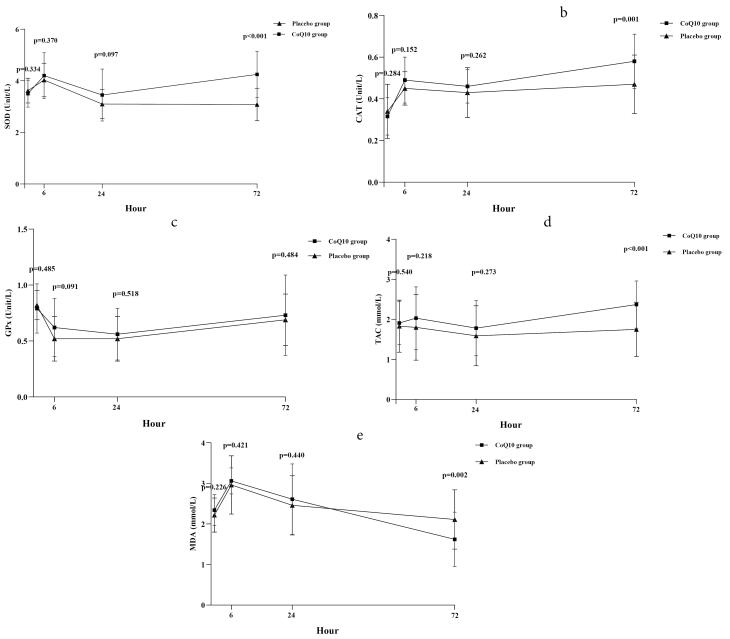
Time-concentration curve of: a. superoxide dismutase (SOD); b. catalase (CAT); c. glutathione peroxidase (GPx); d. total antioxidant capacity (TAC); e. malondialdehyde (MDA). Data are presented as mean and standard error and were analyzed using the Repeated Measures ANOVA test

 Results regarding the comparison of the plasma level of oxidative stress biomarkers at different study time points between the groups are shown in Figure 2 and more detailed information is provided in Table 2.

**Table 2:** Comparison of the serum levels of oxidative stress biomarkers at different study time points between the study groups

**Variables **		**CoQ10 group (n=35)**	**Placebo group ** **(n=35)**	**Mean difference** **(95% confidence interval)**	***P*** ** value**
SOD (Unit/L)	Before PPCI 6 hour 24 hour 72 hour	3.50 ± 0.52 4.20 ± 0.88 3.45 ± 1.01 4.17 ± 0.82	3.62 ± 0.48 4.03 ± 0.65 3.08 ± 0.78 3.14 ± 0.53	-0.12 (-0.36,0.12) 0.17 (-0.20,0.54) 0.36 (-0.07,0.79) 1.03 (0.71,1.36)	0.334 0.370 0.097 < 0.001
GPX (Unit/L)	Before PPCI 6 hour 24 hour 72 hour	0.79 ± 0.22 0.62 ± 0.26 0.56 ± 0.22 0.73 ± 0.35	0.82 ± 0.13 0.52 ± 0.20 0.52 ± 0.29 0.69 ± 0.23	-0.03 (-0.12,0.05) -0.09 (-0.02,0.20) 0.04 (-0.08,0.17) 0.05 (-0.09,0.20)	0.485 0.091 0.518 0.484
CAT (Unit/L)	Before PPCI 6 hour 24 hour 72 hour	0.32 ± 0.09 0.49 ± 0.11 0.46 ± 0.08 0.58 ± 0.14	0.34 ± 0.13 0.45 ± 0.09 0.43 ± 0.11 0.47 ± 0.14	-0.03 (-0.08,0.03) 0.04 (-0.01,0.08) 0.03 (-0.02,0.07) 0.11 (0.05,0.17)	0.284 0.152 0.262 0.001
TAC (mmol/L)	Before PPCI 6 hour 24 hour 72 hour	1.92 ± 0.54 2.08 ± 0.72 1.78 ± 0.72 2.37 ± 0.59	1.82 ± 0.65 1.85 ± 0.82 1.59 ± 0.75 1.76 ± 0.69	0.09 (-0.20,0.38) 0.23 (-0.13,0.60) 0.19 (-0.15,0.55) 0.62 (0.31,0.93)	0.540 0.218 0.273 < 0.001
MDA (mmol/L)	Before PPCI 6 hour 24 hour 72 hour	2.34 ± 0.39 3.06 ± 0.32 2.61 ± 0.87 1.62 ± 0.67	2.22 ± 0.42 2.96 ± 0.67 2.46 ± 0.73 2.16 ± 0.73	0.12 (-0.08,0.31) 0.10 (-0.15,0.35) 0.15 (-0.23,0.53) -0.53 (-0.87,-0.20)	0.226 0.421 0.440 0.002

*Note:* CoQ10 = Coenzyme Q10; PPCI = primary percutaneous coronary intervention; SOD = Superoxide dismutase; CAT = Catalase activity; GPX = Glutathione peroxidase; TAC = Total antioxidant capacity; MDA = Malondialdehyde; CI = confidence interval. Data were analyzed using the student t-test.*P* < 0.05 statistically significant.

###  Changes in plasma activity of SOD, CAT, GPx activity 

 The changes in plasma SOD activity at different study time points are depicted in Figure 2a. Before PPCI, there was no significant difference in plasma SOD activity between the groups (*P* value = 0.334). At 6 hours post-PPCI, both groups exhibited an increase in mean plasma SOD activity, with no significant difference observed between them (*P* value = 0.370). By the 24-hour, the mean plasma SOD activity in both groups showed a decreasing trend, and there was no significant difference between the groups (*P* value = 0.097). However, at the 72-hour, the mean plasma SOD activity increased in both groups, with a higher increase observed in the CoQ10 group compared to the placebo group. The mean treatment difference was 1.03 (95% confidence interval [CI] = 0.71, 1.36; *P* value < 0.001), indicating a statistically significant difference favoring the CoQ10 group in terms of increased plasma SOD activity. The changes in plasma CAT activity closely paralleled those observed in the SOD activity (Figure 2b). The mean plasma CAT activity demonstrated similarity between the groups before PPCI (*P *value = 0.284). At 6 hours post-PPCI, both groups exhibited an increase in mean plasma CAT activity, and when comparing the activity between the groups, no significant difference was noted (*P *value = 0.152). By the 24-hour, the mean plasma CAT activity decreased slightly in both groups, with no significant difference between the study groups (*P *value = 0.262). At the 72-hour, a trend of increasing mean plasma CAT activity was observed in both groups, yet the rise in plasma CAT activity was more pronounced in the CoQ10 group than in the placebo group. The mean treatment difference was 0.11 (95% CI = 0.05, 0.17; *P *value = 0.001), signifying a statistically significant difference between the groups. In contrast, the changes in plasma GPx activity diverged from those in SOD and CAT activities (Figure 2c). Before PPCI, plasma GPx activity was similar between the groups (*P* value = 0.485). At 6 hours post-PPCI, the mean plasma GPx activity decreased in both groups, and the reduction was similar between the two groups (*P *value = 0.091). At 24 and 72 hours, compared to the 6-hour, mean plasma GPx activity showed a slight upward trend in both groups, with no significant difference between the groups at these time points (*P *value = 0.518 and 0.484, respectively).

###  Changes in Plasma TAC level

 The changes in the plasma level of TAC at different study time points are depicted in Figure 2d. There was no significant difference between the groups in the mean plasma level of TAC before PPCI (*P *value = 0.540). Following PPCI completion, both groups exhibited a decreasing trend in the plasma TAC level at 6 and 24 hours, while showing an increasing trend at 72 hours. When comparing the mean plasma TAC level between the groups at 6 and 24 hours after PPCI, no significant difference was observed (*P *value = 0.218 and 0.273, respectively). However, at 72 hours, the plasma TAC level was significantly higher in the CoQ10-treated group compared to the placebo-treated group, with a mean treatment difference of 0.62 (95% CI = 0.31, 0.93; *P* value < 0.001).

###  Changes in Plasma MDA level

 The changes in the plasma level of MDA at different study time points are illustrated in Figure 2e. The plasma MDA level before PPCI was similar in the two groups (*P* value = 0.226). At 6 hours post-completion of PPCI, both groups exhibited an increasing trend in the plasma MDA level, with no significant difference between them (*P *value = 0.421). However, at 24 and 72 hours after PPCI, the plasma MDA level in both groups showed a decreasing trend. When comparing the mean plasma level of MDA between the study groups at 6 and 24 hours, there was no significant difference (*P *value = 0.440). However, at 72 hours, the plasma MDA level was significantly lower in the CoQ10-treated group compared to the placebo-treated group, with a mean treatment difference of -0.53 (95% CI = -0.87, -0.20; *P *value = 0.002).

###  Comparison of incidence of short-term MACE between the two groups

 Over the 28-day follow-up period, MACE occurred in 2 (5.70%) patients in the intervention group, involving one case of cardiovascular mortality and one case of hospitalization due to heart failure. In the control group, MACE occurred in 5 patients (14.30%), involving two cases of cardiovascular mortality, two cases of hospitalization due to heart failure, and one case of nonfatal myocardial infarction. Despite these variations, no significant difference was observed in the occurrence of MACE between the two groups during the follow-up period (*P *value = 0.428).

## Discussion

 In this study, we investigated the impact of CoQ10 supplementation on plasma levels of oxidant and antioxidant biomarkers after reperfusion therapy in patients with STEMI undergoing PPCI. No significant differences were found between the groups. However, at the 72-hour, the CoQ10-treated group demonstrated significantly higher plasma levels of CAT, SOD, and TAC, along with a lower plasma level of MDA compared to the placebo group. The plasma activity of GPX did not differ between the study groups at the study time points. Additionally, there was no discernible difference between the two groups in the incidence of MACE throughout the 28-day follow-up period.

 It is well known that oxidative stress plays an important role in the pathogenesis of myocardial ischemia-reperfusion injury.^6^ The abrupt reintroduction of high oxygen tensions immediately following reperfusion therapy evokes a burst of ROS production, leading to mitochondrial damage. Subsequent dysfunction of the mitochondrial electron transport chain results in an escalation of toxic ROS formation, overwhelming the endogenous antioxidant defense system.^39^ Antioxidant enzymes, including SOD, CAT, and GPX, act as the first line of antioxidant defense mechanisms against oxidative damage.^40^ SOD facilitates the conversion of the highly reactive superoxide anion to oxygen and hydrogen peroxide (H_2_O_2_), while CAT and GPX are actively involved in the detoxification of hydrogen peroxide.^41^ A decline in the activity of these enzymes is associated with the transformation of H_2_O_2_ into a more reactive HO^•^.^42^ The non-enzymatic antioxidants, including a variety of endogenous biological molecules and exogenous antioxidants, act as a second-line defense against ROS.^43^ TAC is used to measure the overall non-enzymatic antioxidant capacity of the body.^44^ In a high oxidative stress milieu, excessive ROS can cause cellular damage both directly, by destroying proteins, DNA, lipids, and other macromolecules, and indirectly, by triggering other pathological processes, such as apoptosis, autophagy, necrosis, inflammation, and fibrosis.^45^ MDA, a product of lipid peroxidation, serves as an indicator of oxidative stress^46^

 The use of antioxidants, as an effective cardioprotective approach to prevent myocardial ischemia-reperfusion injury has been extensively investigated, yielding encouraging results ^11^ It is believed that antioxidants exert their protective effects through various mechanisms, including inhibiting the formation of ROS, scavenging ROS or their precursors, enhancing endogenous antioxidant defense systems, reducing the catalysis of ROS production via binding to metals ions, and attenuating apoptotic cell death by up-regulating the anti-death gene can diminish ROS-mediated cellular injury.^47^ The potential cardioprotective role of CoQ10 has undergone extensive investigation in experimental models of myocardial ischemia-reperfusion injury, yielding promising results. For example, in a study conducted by Liang et al. in a rat model of acute ischemia-reperfusion injury, CoQ10 supplementation regulated the balance between antioxidants and oxidants, reduced myocardial apoptosis and death, enhanced autophagy, and improved cardiac function^24^ Other studies also support the protective effects of CoQ10, demonstrating its ability to safeguard against apoptotic rat hearts and protect ischemic heart muscle.^48,49^ Notably, a recent meta-analysis of these experimental studies suggests that exogenous CoQ10 administration can significantly decrease myocardial infarct size and improve cardiac function parameters.^28^ Considering the promising preclinical data, the cardioprotective effects of CoQ10 against ischemia-reperfusion injury have been explored in some clinical studies. A prospective study indicated a gradual decrease in serum CoQ10 concentrations after PPCI in STEMI patients. In this study, higher CoQ10 levels at one month post-PPCI were associated with improved left ventricular function during a 6-month follow-up, suggesting a potential predictive role.^27^ The study also linked CoQ10’s cardioprotective effects to its antioxidant and anti-inflammatory properties, with higher CoQ10 levels associated with lower oxidative stress and inflammatory status. ^27^ Some clinical studies also supported early CoQ10 administration in AMI patients, reporting reductions in total cardiac events, including arrhythmias and poor left ventricular function.^26,50^ In our earlier publication, which is integral to the current study, we also found that CoQ10 supplementation could potentially reduce myocardial ischemia-reperfusion injury after PPCI and help to preserve left ventricular function.^30^ Results of one study showed that CoQ10 therapy in patients with AMI is associated with elevated plasma levels of endogenous antioxidant capacity and reduced levels of oxidative stress indicators.^50^ However, contrasting results emerge from a small randomized clinical trial focused on AMI patients, where the co-administration of CoQ10 (100 mg/day) and selenium (100 mg/day) over one year did not exhibit significant effects on the incidence of cardiac failure, ventricular arrhythmias, or other clinical outcomes in these patients, possibly due to the trial’s limited statistical power.^51^ Positive outcomes have been documented in cardiac surgery patients, with a meta-analysis suggesting that preoperative CoQ10 treatment, by enhancing mitochondrial respiration and increasing myocardial tolerance to oxidative stress, could potentially reduce reperfusion arrhythmia and inotropic requirements in patients undergoing CABG surgery.^52^ In elective percutaneous coronary intervention (PCI), a randomized clinical trial showed that a single 300 mg loading dose of CoQ10 before the procedure did not significantly affect CK-MB and TnI levels but led to a significant reduction in high-sensitivity C-reactive protein (hs-CRP) levels. The researchers suggested that a longer treatment duration might be necessary for CoQ10 to fully exert its cardioprotective benefits.^53^

 The cardioprotective effects of CoQ10 during myocardial ischemia-reperfusion injury are likely attributed to various mechanisms, with its antioxidant capacity being a widely recognized primary benefit.^28^ Research suggests that its active antioxidant form, ubiquinol, is crucial for this function.^54^ A study on lipid peroxidation revealed that in the early stages of oxidation processes, ubiquinol acts as a highly effective antioxidant, protecting cell membrane lipids and circulatory lipoproteins.^55^ CoQ10 exhibits both direct and indirect antioxidant properties. Directly, it interacts with and scavenges free radicals. Indirectly, as part of the mitochondrial respiratory chain, CoQ10 inhibits endogenous ROS production within mitochondria, recycles antioxidants like vitamin C and E,^56^, and enhances the activity of antioxidant enzymes (GPX, CAT, SOD) by absorbing free radicals and increasing their gene expression.^57,58^ CoQ10 may also positively influence the non-enzymatic component of the antioxidant defense system by modifying the balance between ROS and antioxidant defense systems.^59,60^ Therefore, CoQ10 can enhances both enzymatic and non-enzymatic antioxidant defense systems. Supporting this perspective, a recent systematic review and meta-analysis of randomized controlled clinical trials involving healthy subjects and patients with various pathological conditions revealed that CoQ10 supplementation increases plasma levels of TAC, enhances the activity of antioxidant enzymes such as SOD, CAT, and GPX, and reduces levels of MDA.^61^ Results from another meta-analysis focused on randomized controlled clinical trials involving patients with coronary artery diseases revealed that CoQ10 supplementation markedly boosts the activity of SOD and CAT, concurrently reducing plasma levels of MDA in this patient group. Notably, there was no substantial impact on GPX activity.^29^ In the present study, we also found that the serum activity of GPX does not significantly change with CoQ10 treatment. Thus, it appears that CoQ10 treatment may not significantly affect glutathione concentration and GPx activity in patients with coronary artery diseases. However, some studies have reported that the CoQ10 treatment did not affect the TAC and MDA levels^62,63^ and the activity of antioxidant enzymes.^64,65^ The observed discrepancies in study outcomes may be attributed to several potential factors, including variations in sample sizes and follow-up periods, the diverse range of disease types, disparities in patients’ health status, and other individual characteristics such as gender and age. Furthermore, the dosage and duration of CoQ10 administration, the formulation type of the supplement, and baseline levels of oxidative stress status are additional factors that could contribute to the conflicting results. Notably, among these factors, the dosage and duration of therapy have been recognized as particularly significant determinants influencing the efficacy of CoQ10 treatment. The effectiveness of CoQ10 supplementation is contingent upon achieving therapeutic serum levels. Higher doses of CoQ10 facilitate the attainment of effective CoQ10 levels in the plasma and all tissues, including the heart.^66^ In our present study, a significant change in oxidative stress biomarkers was observed after 72 hours of CoQ10 treatment, suggesting a likely increase in serum CoQ10 concentration. A study in patients with coronary artery disease similarly indicated that higher doses of CoQ10 may result in faster antioxidant effects.^67^ One notable property of CoQ10 that renders it an ideal dietary supplement is its excellent safety profile, even with chronic exposure to high doses.^68^ CoQ10’s excellent safety profile allows for the use of high doses in disease treatment without adverse side effects.

## Limitations

 This study has several limitations: a small participant number due to single-center design and strict inclusion/exclusion criteria, reducing statistical power for MACE incidence but sufficient for detecting differences in oxidative stress biomarkers between the groups. The strict inclusion and exclusion criteria resulted in a cohort limited to highly selective STEMI patients undergoing reperfusion therapy, thereby restricting the generalizability of our findings to all STEMI patients. Constraints on research grants prevented assessing CoQ10 effects on serum levels of other systemic oxidative stress biomarkers, such as nitric oxide (NO) as a nitrosative stress indicator. The measurement of plasma oxidative stress biomarkers was conducted solely within the initial 72 hours after myocardial reperfusion. However, their value during this period can be extrapolated and considered as an estimated indicator of the subsequent oxidative stress status. Concurrent standard PPCI treatment could influence the assessment of the CoQ10’s true effect on oxidative stress biomarkers, although medication use was comparable between groups. Given these limitations, further studies with larger sample sizes and longer follow-up periods are necessary to validate the beneficial effects of CoQ10 and to comprehensively evaluate its impact on various oxidative stress biomarkers, as well as other cardioprotective mechanisms implicated in ischemia-reperfusion injury.

## Conclusion

 In this study, we observed that administering CoQ10 to patients with STEMI undergoing PPCI, a dose of 400 mg before PPCI and 200 mg twice a day for three days after PPCI, is associated with an increase in plasma levels of antioxidants biomarkers, including SOD, CAT, and TAC and decrease in oxidative stress assessed by MDA levels, at 72 hours after PPCI. Considering the important role of oxidative stress in myocardial ischemia-reperfusion injury, these findings suggest that CoQ10 administration may reduce the reperfusion injury of ischemic myocardium in patients with STEMI undergoing PPCI by regulating the balance between antioxidant and oxidant biomarkers during reperfusion therapy. However, to confirm this effect and elucidate other mechanisms by which CoQ10 exerts its cardioprotective effects in this patient population, additional high-quality randomized controlled trials are warranted.

## Acknowledgments

 The authors express their sincere appreciation to the all participants and medical staff of the Farshchian Cardiovascular Hospital for contributing in this study.

## Competing Interests

 None of the authors of this article have any conflicts of interest to declare.

## Ethical Approval

 The trial protocol was according to the Declaration of Helsinki as revised in 1989, and the study protocol was approved by the research and ethics committee of the Hamadan University of Medical Sciences, (IR.UMSHA.REC.1400.623). The participants provided their written informed consent to participate in this study.

## Funding

 This research was financially supported by the vice-chancellor for research and technology, Hamadan University of Medical Sciences, Hamadan, Iran (No: 140009167713). This grant was not assigned to the manuscript writing, editing, and publication fee.
